# Decoding MRI-informed brain age using mutual information

**DOI:** 10.1186/s13244-024-01791-9

**Published:** 2024-08-26

**Authors:** Jing Li, Linda Chiu Wa Lam, Hanna Lu

**Affiliations:** 1 Department of Psychiatry, The Chinese University of Hong Kong, Hong Kong SAR, China; 2 The Affiliated Brain Hospital of Guangzhou Medical University, Guangzhou, China

**Keywords:** Brain age, Structural MRI, Mutual information, Machine learning, Gray matter volume

## Abstract

**Objective:**

We aimed to develop a standardized method to investigate the relationship between estimated brain age and regional morphometric features, meeting the criteria for simplicity, generalization, and intuitive interpretability.

**Methods:**

We utilized T1-weighted magnetic resonance imaging (MRI) data from the Cambridge Centre for Ageing and Neuroscience project (*N* = 609) and employed a support vector regression method to train a brain age model. The pre-trained brain age model was applied to the dataset of the brain development project (*N* = 547). Kraskov (KSG) estimator was used to compute the mutual information (MI) value between brain age and regional morphometric features, including gray matter volume (GMV), white matter volume (WMV), cerebrospinal fluid (CSF) volume, and cortical thickness (CT).

**Results:**

Among four types of brain features, GMV had the highest MI value (8.71), peaking in the pre-central gyrus (0.69). CSF volume was ranked second (7.76), with the highest MI value in the cingulate (0.87). CT was ranked third (6.22), with the highest MI value in superior temporal gyrus (0.53). WMV had the lowest MI value (4.59), with the insula showing the highest MI value (0.53). For brain parenchyma, the volume of the superior frontal gyrus exhibited the highest MI value (0.80).

**Conclusion:**

This is the first demonstration that MI value between estimated brain age and morphometric features may serve as a benchmark for assessing the regional contributions to estimated brain age. Our findings highlighted that both GMV and CSF are the key features that determined the estimated brain age, which may add value to existing computational models of brain age.

**Critical relevance statement:**

Mutual information (MI) analysis reveals gray matter volume (GMV) and cerebrospinal fluid (CSF) volume as pivotal in computing individuals’ brain age.

**Key Points:**

- Mutual information (MI) interprets estimated brain age with morphometric features.
- Gray matter volume in the pre-central gyrus has the highest MI value for estimated brain age.
- Cerebrospinal fluid volume in the cingulate has the highest MI value.
- Regarding brain parenchymal volume, the superior frontal gyrus has the highest MI value.
- The value of mutual information underscores the key brain regions related to brain age.

**Graphical Abstract:**

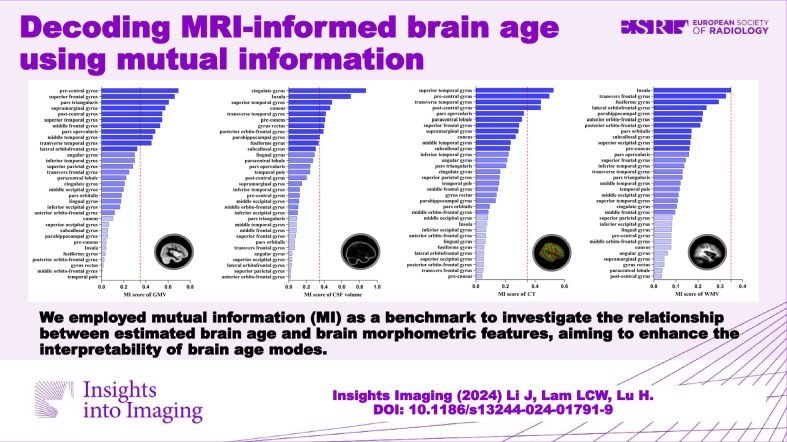

## Introduction

Aging, as an inevitable and irreversible process, manifests uniquely in each individual and is influenced by a complex interplay of genetic predisposition [1, 2], environmental determinants [3], lifestyle choices [1], and neural plasticity [2]. As individuals progress through the chronological trajectory of life, their brains undergo a series of intricate changes. These brain changes encompass a wide spectrum of physiological and pathological processes, ranging from subtle modifications in cortical layers to region-specific volumetric variations [3, 4], culminating in distinctive patterns of age-related brain morphometry. The structural changes in the brain manifest in a multidimensional manner, impacting both volumetric and surface-based features [5].

With the development of quantitative approaches in neuroimaging, the concept of brain age has been developed for thoroughly estimating an individual’s chronological age at individual level [6]. Structural magnetic resonance imaging (sMRI) plays a pivotal role in capturing the subtle yet crucial changes in brain morphometry that unfold over an individual’s lifespan. The utilization of sMRI combined with advanced computational techniques, such as machine learning algorithms, empowers neuroscientists to develop predictive models linking brain structures with chronological age [7]. Estimated brain age has the potential to identify deviations from the norms, shedding light on understanding accelerated or decelerated brain aging trajectories [8]. The difference between predicted brain age and chronological age is referred to as the score of brain-PAD (brain-predicted age difference) [9]. For example, when considering an individual with a chronological age of 60 years and a predicted brain of 65 years, the score of brain-PAD is +5 years, indicating that the individual’s brain is undergoing an accelerated aging process of +5 years.

The brain age model has shown promising utilities in clinical populations. For example, brain age prediction can be used to identify abnormal aging-related deviations in individuals with psychiatric and neurological disorders, such as schizophrenia and Alzheimer’s disease [10–14]. Of note, these deviations from normal aging trajectory can be detected before clinical symptoms appear, allowing for earlier intervention and potentially improving clinical outcomes [7, 13–15]. The estimated brain age may also serve as a biomarker of an individual’s brain health during aging [7]. By estimating an individual’s brain age, clinicians can gain insights into their cognitive health and the risk of developing age-related conditions [12, 15, 16].

Despite the promise of brain age, it encounters challenges, notably two ‘black boxes’. First, the value of brain age is overly a ‘black box’ [7]. Brain age, estimated from structural brain scans, aggregates the complexities underlying multidimensional alteration patterns of brain aging into one value [16]. Age-related alterations in the brain are subtle, nonlinear, and spatially distributed [17–19]. Although brain age has been widely studied in psychiatric and neurological disorders, the specific features used to predict brain age remain unclear, which may result in disregarding important neuroscientific information [7]. This is of particular concern for researchers to extend the utilities of brain age to conditions whose pathoanatomical deviations from typical aging may feature both global and regional changes. Second, brain age is estimated by utilizing various statistical models, including classical statistical models (e.g., multivariate and univariate regression, general linear models), machine learning models (ML) (e.g., random forest regression, elastic net regression, relevance vector regression), and deep learning techniques (DL) (e.g., convolutional neural networks) [20–25]. It should be noted that most ML and DL techniques are ‘black boxes’ [26]. Unlike traditional statistical methods, where the parameters and assumptions of the models are explicitly defined, the models computed by ML and DL algorithms are operated by optimizing parameters through iterative processes, resulting in complex decision boundaries that are very challenging to interpret. The specific features or patterns identified by the ML and DL models may not be readily interpretable and understood. The ‘black box’ nature of ML and DL models raises concerns about their reliability and accountability when applied to brain age prediction. The lack of interpretability in these models may limit the applications of brain age in clinical populations.

To address these concerns, we employed mutual information (MI) value as a standardized metric to quantify the distinct contributions of morphometric features to estimated brain age. MI is a fundamental notion in information theory, quantifying the extent to which one random variable holds information about another, which is widely employed to gauge the statistical relatedness of any relationship between variables [27]. This property allows MI value to reliably measure the statistical dependency between variables without being affected by transformations, thereby providing a robust and consistent measure of the relationship between estimated brain age and morphometric features across different representations or coordinate systems. The MI value of individual’s variables also remains invariant under any invertible transformations [28]. Thus, using MI value to gauge the impact of input morphometric features on brain age has three-fold advantages: (1) simplicity in computation, avoiding resource-intensive procedures, especially when dealing with complex models and large datasets; (2) generalizability well across different types of statistical models by capturing the feature patterns in any model; (3) intuitive interpretability, providing understandable insights with minimal needs for statistical assumptions or domain-specific expertise. In this study, we initially employed volumetric- and surface-based brain regional features as training features to develop a brain age model. Subsequently, we utilized the nearest-neighbor method to compute the MI values between each input feature and the estimated brain age [27, 29]. This approach aimed to bridge the gap in the interpretability of brain age models and facilitate the validation of model predictions, thereby enhancing the credibility of brain age estimation. Furthermore, it may advance our understanding of the relationship between brain aging and macroscopic structural changes in aging populations.

## Materials and methods

### Participants

The training set (*N* = 609, aged from 18 to 88 years) comprising T1-weighted structural MRI scans was obtained from the Cambridge Centre for Aging and Neuroscience (Cam-CAN) study (https://www.cam-can.org) [30]. The testing set (*N* = 547, aged from 20 to 86 years) comprised T1-weighted sMRI data from the Brain development project (IXI) (https://brain-development.org). The demographic characteristics of the participants in the two datasets, along with the MRI acquisition parameters, are listed in Table 1. As per local study protocols [30], all participants underwent screening to ensure cognitive health and exclude major psychiatric conditions. The testing set served for both validation of the brain age prediction model and quantification of individual feature contributions to the estimated brain age.

**Table 1:** The detailed information of Cam-CAN and Brain development project

	Cam-CAN	Brain development project (IXI)
Number of subjects	609	547
Males/females	305/304	243/304
Age mean (SD)	53.2 (18.3)	48.6 (16.5)
Age range	18–88	19.98–86.32
TR (ms)	2250	9.6/9.8
TE (ms)	2.99	4.6/4.6
TI (ms)	900	\
FOV	256 × 240 × 192	\

*Cam-CAN* Cambridge Centre for Aging and Neuroscience, *TR* repetition time, *TE* echo time, *TI* inversion time, *FOV* field of view, *SD* Standard deviation

### Pre-processing of sMRI scans

Cortical reconstructions and surface-based morphometry analysis of T1-weighted MRI scans were conducted using BrainSuite 21a (https://brainsuite.org/) [31]. BrainSuite is a semi-automatic cortical surface identification integrated package, widely employed in aging research [32, 33]. The pre-processing pipeline encompassed the following steps: (1) cortical surface extraction involves several steps: skull-stripping, tissue classification, gross labeling of brain structures, and modeling the inner and outer boundaries of the cerebral cortex. Notably, image enhancement and pre-processing techniques are applied before the skull-stripping process. An anisotropic diffusion filter is applied to smooth contiguous tissue regions while respecting the edge boundaries between them, enhancing the boundary between the brain and other tissues [34]. A three-dimensional Marr Hildreth edge detector and morphological erosion processing are then utilized to remove the skull and scalp from the image [35, 36]. Image nonuniformities are corrected in the stripped brain, using a parametric tissue measurement model [31]; (2) cortical thickness estimation based on partial volume estimates and anisotropic diffusion equation; (3) surface-constrained volumetric registration to create a mapping to a labeled reference atlas (BCI-DNI) [37] and assign labels to the cortical surface and brain volume; (4) alignment of cortical thickness estimates to atlas space; (5) computation of subject-level features, including gray matter volume (GMV), white matter volume (WMV), cerebrospinal fluid (CSF) volume, and cortical thickness in the pre-defined regions of interest. Visual inspections were performed, and any segmentation errors were manually corrected.

### Regional cortical features

We utilized four types of regional morphometric features, including GMV, WMV, CSF volume, and cortical thickness, in total 66, as input features for the brain age model. Given the anatomical asymmetry of the brain, we extracted regional morphometric features from both left and right brain hemispheres [38–40]. A total of 66 cortical regions, covering both hemispheres, were chosen according to the BCI-DNI atlas. To systematically explore the impact of regional morphometric features on estimated brain age, we investigated the combinations of different morphometric features for each cortical region, including 66 regional brain parenchyma volume features, merging the GMV and WMV of each cortical region; and 66 regional intracranial volume features, incorporating the GMV, WMV, and CSF volumes of each cortical region.

### Support vector regression model

We utilized support vector regression (SVR), a robust regression model implemented with the scikit-learn library in Python, widely used for estimating brain age [20, 41–46]. SVR seeks to identify a hyperplane that minimizes deviation from training data, skin to linear regression [47]. Unlike linear regression, SVR could compute errors solely from data points beyond a ‘margin of tolerance’ set by the hyperparameter epsilon ($\epsilon$), known as support vectors, which dictate the hyperplane’s placement. The regularization hyperparameter ‘C’ plays a vital role in striking a balance between hyperplane complexity and training errors, effectively preventing overfitting. We utilized the radial basis function (RBF) kernel to facilitate mapping nonlinear data into higher dimensions through the ‘kernel trick’. We applied a nested 10-fold cross-validation scheme and GridSearchCV function with the ‘neg_mean_absolute_error’ scoring parameter to determine the optimal regularization hyperparameters for SVR. We then used the ‘best_params_ ‘attribute to obtain the value of the regularization hyperparameter ‘C’, resulting in C = 1.

### Age-bias correction

In the estimation of brain age, a common phenomenon is age-related bias, with younger individuals often having overestimated brain ages and older individuals having underestimated brain ages due to general statistical features of the regression analysis [41, 48, 49]. To address this age-related bias, we employed a statistical age-bias correction method. This method involves fitting the estimated brain age into a regression function:

$$\hat{{{corrected\; age}}_{i}}=\hat{{{age}}_{i}}+[{{age}}_{i}-\left(\alpha \times {{age}}_{i}+\beta \right)]$$

In this equation, $\hat{{{corrected\; age}}_{i}}$ represents the corrected brain age for subject $i$, $\hat{{{age}}_{i}}$ is the first estimated brain age, and ${{age}}_{i}$ is the chronological age of subject $i$. The correction coefficients *α* and *β* are estimated through a fit in the training set and subsequently applied to correct estimations in the testing set [50].

### Decoding the contributions of brain regions

Mutual information (MI) gauges the interdependence of variables, first defined and analyzed by Shannon in 1948 [51]. MI is based on the concept of entropy, which measures the uncertainty or randomness of a random variable. The calculation of entropy is based on the probability function of the random variable, where higher entropy indicates greater uncertainty and information. For a random variable, $x$, its entropy, denoted by $H\left(x\right)$, represents the uncertainty associated with $x.$ The entropy of $x$ is:

$$H\left(x\right)=-{\sum}_{i=1}^{n}p\left(\right.x\left(i\right)\cdot \log (x\left(i\right))$$

$x\left(i\right)$) represents each possible outcome of the random variable $x.$ $P(x\left(i\right))$ is the marginal probability of $x\left(i\right)$). For two variables, $x$ and $y$, their MI: $I\left({x;y}\right)$ quantifies the reduction in uncertainty about one variable when the other variable is known. Mathematically, MI can be expressed as the difference between the joint entropy of $x$ and $y$, and the sum of their individual entropies:

$$I\left(x{{{\rm{;}}}}\, y\right) = H\left(x\right)+H\left(y\right)-H(x,y)$$

$$I\left(x{{{\rm{;}}}}\, y\right) = {\sum}_{i=1}^{n}{\sum}_{j=1}^{n}p\left(x\left(i\right),y\left(j\right)\right)\left(\frac{p\left(x\left(i\right),y\left(j\right)\right)}{p\left(x\left(i\right)\right)\cdot p\left(y\left(j\right)\right)}\right)$$

MI is non-negative ($I\left({x;y}\right)\ge 0$) and symmetric ($I\left({x;y}\right)=I\left({y;x}\right)$). A MI value of zero indicates that the two variables are independent, meaning the knowledge of one variable does not provide any information about the other variable. A higher MI value signifies stronger dependencies between variables. In this study, we applied the KSG estimator proposed by Kraskov, Stögbauer, and Grassberger for computing the MI [27]. The KSG method involves an adaptive non-parametric approach that estimates MI without making assumptions about the underlying probability distributions. It utilizes a k-nearest neighbor to estimate local densities and adaptive bin the data. The KSG method offers advantages such as robustness to noise and adaptiveness to data structure. Below is the equation:

$$I\left(X,Y\right)=\psi \left(k\right)-\left\langle \psi \left({n}_{x}+1\right)+\psi \left({n}_{y}+1\right)\right\rangle +\psi \left(N\right)$$

$N$ is the number of data points in the joint space.

$k$ is the number of nearest neighbors.

$\psi$ is the digamma function.

${n}_{x}$ and ${n}_{y}$ are neighbor counts within X and Y marginal spaces.

## Results

### Model performance

The brain age model was trained using 264 training features, encompassing mean cortical thickness, regional volumes of GMV, WMV, and CSF. Three commonly used metrics of model performance were included in this study: (1) root-mean-square error (RMSE) [52], (2) mean absolute error (MAE) [52], and (3) coefficient of determination (${R}^{2}$) [53]. The age-bias correction coefficients (*α* = 0.885, *β* = 5.658) were derived from a fit in the training set and applied to correct predictions in the independent testing set. On the training set, the model reached an accuracy of MAE = 5.32 years, RMSE = 6.64 years, and ${R}^{2}$ = 0.87. After age-bias correction, the performance of the brain age model was improved accordingly (MAE = 5.15 years, RMSE = 6.27 years and ${R}^{2}$ = 0.88).

**Fig. 1:**
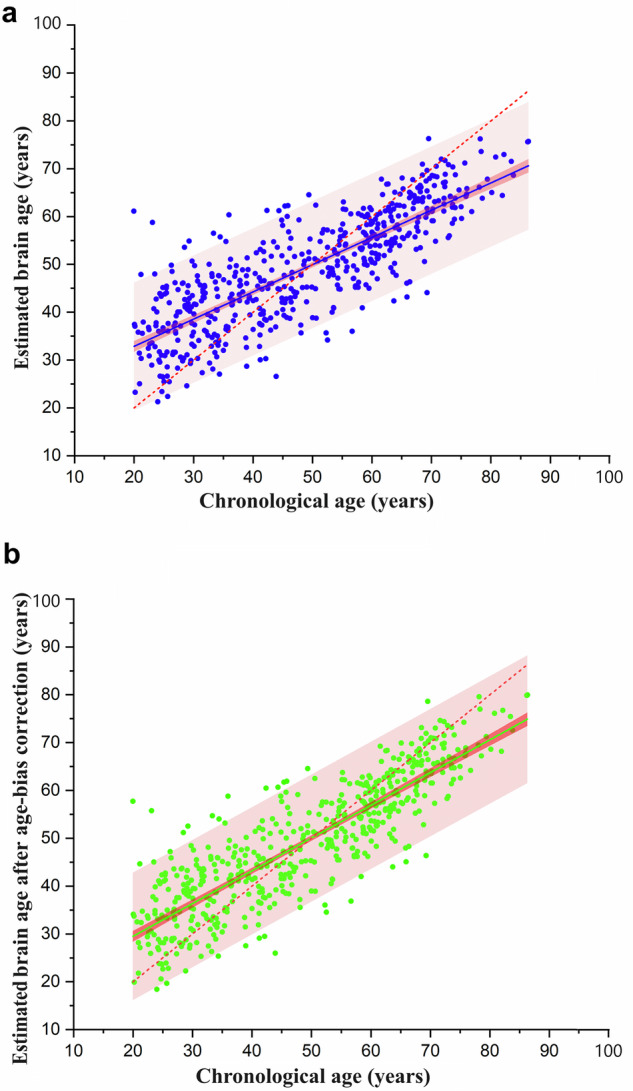
The relationship between estimated brain age and chronological age in the testing set. Prediction bands and 95% confidence bands are presented. **a** presents the relationship between estimated brain age and chronological age. The red line is a regression line fitted when estimated brain age equals chronological age. The blue line is a regression line fitted between estimated brain age and chronological age. **b** displays the relationship between the estimated brain age after age-bias correction. The red line is a regression line fitted when the age-bias-corrected estimated brain age equals chronological age. The green line is a regression line fitted between the estimated brain age after age-bias correction and chronological age

In the independent testing set, the relationship between chronological age and estimated brain age, as well as chronological age and estimated brain age after age-bias correction, were illustrated in Fig. 1. The performance of the brain age model in the testing set had a slight decrease, resulting in MAE = 7.79 years, RMSE = 9.80 years, and ${R}^{2}$ = 0.66. However, after age-bias correction, an improvement in the model’s prediction accuracy was observed: MAE = 6.65 years, RMSE = 8.53 years, and ${R}^{2}$ = 0.74.

### Contributions of different brain features

**Fig. 2:**
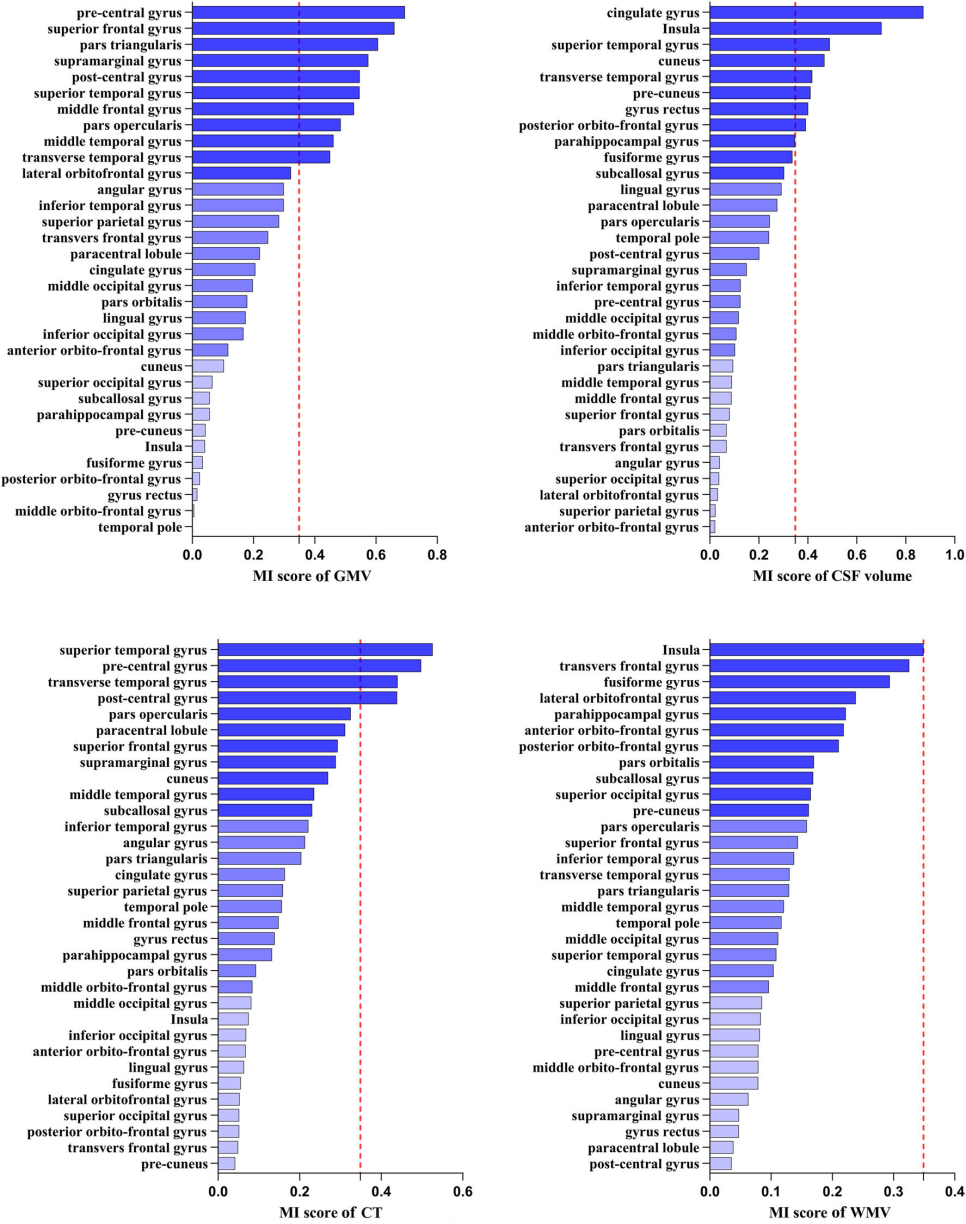
Detailed ranking of mutual information (MI) value between estimated brain age after age-bias correction and four brain morphometric features (GMV, CSF volume, CT, and WMV) across 33 brain regions. Brain regions are classified into three groups based on MI values: the highest one-third, the middle one-third, and the lowest one-third depicted by varying color intensities. A red reference line is provided, indicating an MI value of 0.35, which is the MI between WMV in the insula and estimated brain age after age-bias correction. GMV, gray matter volume; CSF, cerebrospinal fluid; CT, cortical thickness; WMV, white matter volume

**Fig. 3:**
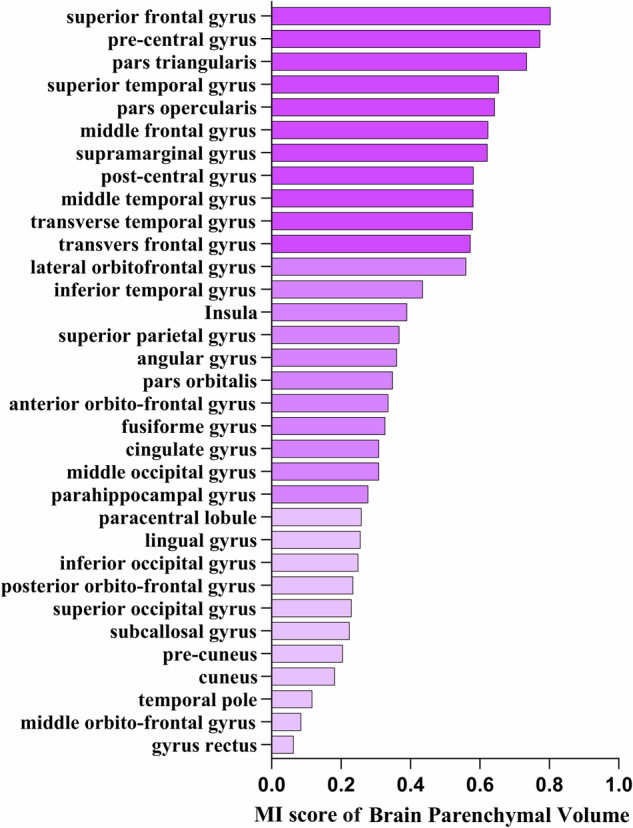
Detailed ranking of mutual information (MI) values between estimated brain age after age-bias correction and brain parenchymal (GMV combined with WMV) features across 33 brain regions. The brain regions are classified into three groups based on MI values: the highest one-third, the middle one-third, and the lowest one-third depicted by varying color intensities

**Fig. 4:**
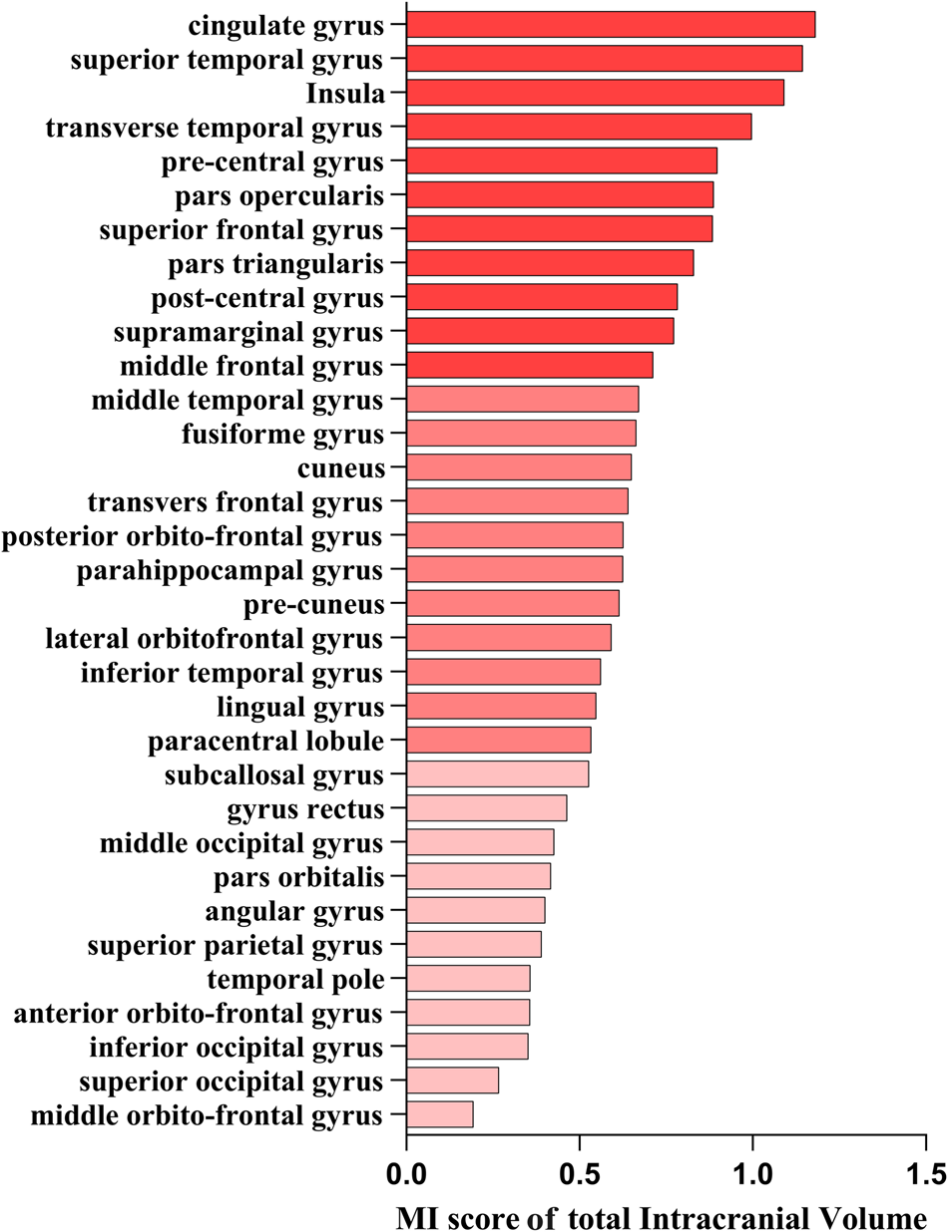
Detailed ranking of mutual information (MI) between estimated brain age after age-bias correction and total intracranial volume (GMV combined with WMV and CSF volume) features across 33 brain regions. These brain regions are classified into three groups based on MI values: the highest one-third, the middle one-third, and the lowest one-third, visually represented by differences in color intensity

**Fig. 5:**
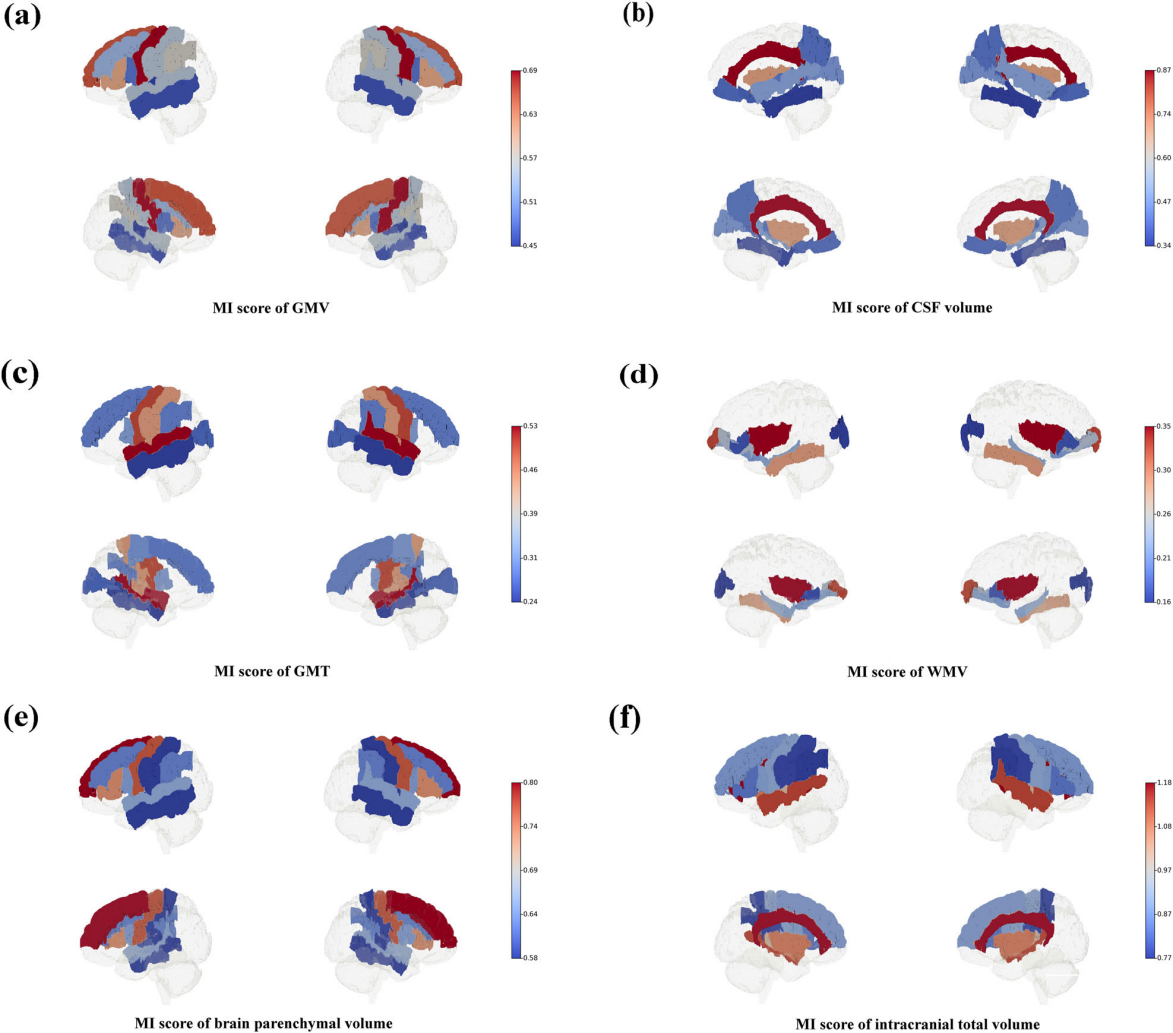
This figure illustrates brain heatmaps showcasing the top ten brain regions in the BCI-DNI atlas, ranked by mutual information (MI) values. **a** shows the top ten regions in gray matter volume (GMV). The pre-central gyrus demonstrates the highest MI value (0.69). **b** illustrates the top ten regions in cerebrospinal fluid (CSF) volume. The cingulate exhibits the highest MI value (0.87). **c** displays the top ten regions in cortical thickness. The superior temporal gyrus exhibits the highest MI value (0.53). **d** presents the top ten regions in white matter volume (WMV). The insula shows the highest MI value (0.35). **e** depicts the top ten regions in brain parenchyma volume (combining GMV and WMV). The superior frontal gyrus displays the highest MI value (0.80) among brain regions. **f** shows the top ten regions in intracranial total volume (the combination of GMV, WMV, and CSF volume). The cingulate showed the highest total MI value (1.18)

The MI values between the estimated brain age and each input brain feature were computed separately. Furthermore, to explore associations between regional brain parenchyma volumes and estimated brain age, we aggregated the MI values of GMV and WMV within the same brain region. To investigate the relationship between regional intracranial volumes and estimated brain age, we aggregated the MI values of GMV, WMV, and CSF volume within each brain region. Geometric asymmetry between brain hemispheres was considered, with input features from both hemispheres included for each brain region. To focus on the overall morphometric characteristics of brain regions. MI values for the same brain region across the hemispheres were merged. The MI values for the 33 cortical brain regions, shown in Figs. 2–4, with the top ten ranked visualized as heatmaps in Fig. 5.

Notably, although the total MI value (27.28) between age-corrected brain age and all features was lower than the total MI value (28.21) between estimated brain age and all features, there were no significant differences in the MI values between age-corrected brain age, estimated brain age, and the features of each brain region. Given that age-bias correction is a commonly employed step in brain age estimations, we discussed the results obtained from the age-corrected brain age to dissect the specific contributions of brain region features. Among the four types of brain features, GMV exhibited the highest total MI value (8.71), with the pre-central gyrus having the highest MI score (0.69) (Fig. 5a). The second-highest total MI value was associated with CSF volume (7.76), with the cingulate having the highest MI score (0.87) (Fig. 5b). The third-highest total MI value corresponded to mean CT (6.22), with the superior temporal gyrus showing the highest MI value (0.53) (Fig. 5c). WMV had the lowest total MI value (4.59), with the highest MI value observed in the insula (0.35) (Fig. 5d). Regarding the brain parenchymal volume (the combination of GMV and WMV), the superior frontal gyrus exhibited the highest total MI value (0.80) (Fig. 5e). In the case of intracranial total volume (the combination of GMV, WMV, and CSF volume), the cingulate showed the highest total MI value (1.18) (Fig. 5f).

### MI in gender-specific testing subgroups

We divided the IXI dataset into female subgroup (sample size: 304; age range: 19.98–86.32 years), and male subgroup (sample size: 243; age range: 20.07–86.20 years). The brain age prediction model performance on the male subgroup is *R*^2^ = 0.68, MAE = 7.74 years, and RMSE = 9.61 years. After age-bias correction, the model performance on the male subgroup is slightly improved: *R*^2^ = 0.75, MAE = 6.67 years, and RMSE = 8.37 years. The brain age prediction model on the female subgroup is *R*^2^ = 0.64, MAE = 7.84 years, and RMSE = 9.96 years, which is less accurate than on the male subgroup. After age-bias correction, the model performance on the female subgroup is slightly improved: *R*^2^ = 0.72, MAE = 6.65 years, and RMSE = 8.66 years. We computed the MI between estimated brain age after age-bias correction and all features in male and female subgroups. The total MI in the male subgroup is 32.65, which is higher than the total MI in the female subgroup (28.58). In the male subgroup, CSF volume exhibited the highest MI (11.62) among the four types of brain features, while GMV exhibited the second-highest MI (8.95). In the female subgroup, GMV exhibited the highest MI (9.78) among the four types of brain features, while CSF volume exhibited the second-highest MI (7.80). We calculated the Spearman ranking correlation coefficient and *p*-value to assess the statistical significance of MI in brain regions between male and female subgroups, and the whole testing dataset. A significance level of 0.05 was set. The results suggested that there was no significant difference in the MI value rankings of each brain region between the female subgroup, the male subgroup, and the overall testing dataset.

## Discussion

In this study, we used four types of morphometric features to train the brain age model. Subsequently, we applied and validated this model to an independent testing dataset to calculate the estimated brain age. Through a comparative analysis of mutual information (MI) values between each input feature, the combinations of input features (brain parenchyma and intracranial total volume), and estimated brain age, we observed several interesting findings. These findings underscore the critical significance of specific MRI-based morphometric features in determining individuals’ brain age and providing new insights into the processes of brain aging through computational models. We used gender-specific subgroups to validate our findings, and the results showed that there were no significant differences in the MI value rankings of each brain region among the gender-specific subgroups.

First and foremost, cortical gray matter volume showed the strongest relatedness with estimated brain age. This observation highlights the superior prediction accuracy of brain age models utilizing gray matter as an input feature compared to other types of morphometric features [54, 55], as evidenced by its widespread applications in the research field of brain age [6, 13, 56–61]. Previous MRI studies have shown age-related anatomical changes in gray matter, including a linear decrease in global gray matter volume (GMV) and regional GMV during aging [62]. As a result, using GMV as an input feature for estimating brain age not only presents its biological plausibility but also makes a substantial contribution to model performance. Additionally, another volumetric feature related to CSF volume exhibited the second-highest correlation coefficient with estimated brain age, following GMV. In line with a previous study [63], this finding also highlights the potential of CSF volume as a valuable feature for brain age estimation. Changes in CSF volume encapsulate information pertinent to brain age. However, it is important to note that the application of CSF-specific features in current studies of brain age remains limited. Second, among the features of brain parenchymal volume, the superior frontal gyrus (SFG) exhibited the strongest association with estimated brain age. From a perspective of neuroanatomy, the SFG is a prominent ridge located on the neocortex, constituting roughly one-third of the frontal lobe. From a perspective of neuropsychology, the core cognitive abilities thought to be dependent on the frontal cortex, such as task-switching, executive functions, verbal fluency, complex attention, and performance monitoring, are particularly vulnerable to the aging process [64–67]. The linkage between neuroanatomy and cognitive functions may offer another unique perspective to understand the feature-specific brain age model. For instance, in 1997, Hannien et al discovered a positive correlation between category verbal fluency test (CVFT) scores and the volume of the frontal lobe [68]. Subsequently, Lu and her colleagues found that neurocognitive impairment (NCD) patients showing older brain age performed significantly worse on CVFT compared to normal aging controls [69]. Thus, our findings might confirm the significance of frontal volume in brain age estimation. When considering brain parenchymal and CSF volume, the total intracranial volume of the cingulate had the highest MI value with brain age, which may highlight the significance of the cingulate gyrus in age-related brain changes. In Alzheimer’s disease (AD), MRI studies consistently report reduced volumes of the cingulate gyrus compared to normal controls [70, 71], with voxel-based morphometric analysis indicating decreased gray matter density, particularly in the posterior regions [72–74]. Reduced metabolic changes (i.e., hypometabolism) in the posterior cingulate gyrus have also been observed in AD patients using magnetic resonance spectroscopy [75] and and positron emission tomography [76–78]. Understanding the changes in the cingulate might help to address cognitive decline in aging and neurodegenerative diseases and further tailor the preventive measures for brain health.

The findings of this study mark a significant and practical advancement for several reasons. First, we incorporated four distinct morphometric features into the brain age model simultaneously, taking a whole-brain perspective. In the field of brain age research, raw MRI scans are commonly used as input features due to their inherent multidimensional morphometric information [54, 79–81]. We improved the interpretability of brain age models by extracting regional morphometric information from MRI scans through quantitative pre-processing, which was utilized as input features. Second, this study explored a novel use of MI as a standardized method to interpret brain age models. MI is an ideal statistic for quantifying the degree of relationship between variables in multiple ways [82]. The rates of brain atrophy vary nonlinearly with age, as observed in the temporal lobe [83] while showing a linear pattern in regions such as the hippocampus and frontal lobe [84, 85]. MI could equally quantify the correlation between each brain region feature and brain age at the individual level. Besides, the nonlinear nature of the relationship between regional morphometric features and brain age may explain why multivariate linear regression models often lack optimal performance in brain age studies. In contrast, support vector machine (SVM), as a commonly used computational model, can effectively capture both linear and nonlinear relationships [45, 86]. Besides, MI provides a straightforward interpretation by measuring the shared information between morphometric features and estimated brain age, grounded on the well-established theoretical framework of information theory. With an advantage of independence, MI is insensitive to the size of the datasets and thus, can converge with tight error bounds to a measure of relatedness [29].

Although our model showed slight overfitting in the testing set, we deliberately avoided using the techniques of dimensionality reduction, such as principal component analysis, or independent component analysis for pre-processing. We retained all the input features during training and testing to maintain the interpretability of brain age estimation and avoid the obfuscation introduced by the techniques of dimensionality reduction. Our objective was to evaluate the interpretability of regional-level features for brain age predictions in an independent testing set rather than pursuing lower MAE. Still, our model’s accuracy is comparable to that of other brain age studies [8, 21].

## Conclusion

Using the mutual information between morphometric features and estimated brain age is a promising way to assess the regional contributions to brain age. The significant roles of frontal gray matter volume and limbic CSF volume highlight that regional gray matter and CSF are key features that determine the estimated brain age, which may add value to existing computational models of brain age.

### Limitations and future directions

The findings of this study have to be interpreted in the context of limitations. First, we used two databases as the training and testing sets, which may limit the generalizability of our results. In future studies, our goal is to incorporate broader demographic databases with larger sample sizes to enhance the robustness of our findings. Second, our brain age model exhibited mild overfitting. We will improve the prediction accuracy and generalization of the pre-trained model while retaining the dimensionality of input features, by increasing the data amount and exploring diverse regression models. Third, our analysis of brain morphometric features was limited to cortical regions. Additionally, we only utilized morphometric features extracted from a single modality of MRI (i.e., structure MRI) as training features in the brain age model. Incorporating multimodal age-related neuroimaging features, such as DTI-based (diffusion tensor imaging) metrics, white matter hyperintensities on T2-weighted MRI, as well as the presence and volume of metals on susceptibility-weighted imaging scans, could provide important supplementary information to the brain age prediction model, thereby enhancing the prediction efficiency of the trained algorithm. Lastly, fine-grained regional brain parcellation is essential for precision targeted therapy. We intend to refine brain parcellation, which will contribute to a more comprehensive analysis and interpretation of brain age in various neurological disorders.

## Data Availability

The data supporting the findings of this study are available on request from the corresponding author. The data are not publicly available due to privacy or ethical restrictions.

## Abbreviations

AD: Alzheimer’s disease
Brain-PAD: Brain-predicted age difference
Cam-CAN: Cambridge Centre for Aging and Neuroscience
CSF: Cerebrospinal fluid
CT: Cortical thickness
CVFT: Category verbal fluency test
DL: Deep learning
GMV: Gray matter volume
MAE: Mean absolute error
MI: Mutual information
ML: Machine learning
RMSE: Root-mean-square error
SFG: Superior frontal gyrus
SVR: Support vector regression
WMV: White matter volume
